# Oral Microbiota Composition and Antimicrobial Antibody Response in Patients with Recurrent Aphthous Stomatitis

**DOI:** 10.3390/microorganisms7120636

**Published:** 2019-12-01

**Authors:** Zuzana Stehlikova, Vojtech Tlaskal, Natalie Galanova, Radka Roubalova, Jakub Kreisinger, Jiri Dvorak, Petra Prochazkova, Klara Kostovcikova, Jirina Bartova, Marketa Libanska, Radka Cermakova, Dagmar Schierova, Antonin Fassmann, Petra Borilova Linhartova, Stepan Coufal, Miloslav Kverka, Lydie Izakovicova-Holla, Jitka Petanova, Helena Tlaskalova-Hogenova, Zuzana Jiraskova Zakostelska

**Affiliations:** 1 Institute of Microbiology of the CAS, v.v.i., 142 20 Prague, Czech Republic; 2 First Faculty of Medicine, Charles University, 121 08 Prague, Czech Republic; 3 Faculty of Science, Charles University, 128 00 Prague, Czech Republic; 4 Department of Zoology, Faculty of Science, Charles University, 128 00 Prague, Czech Republic; 5 Institute of Dental Medicine, Department of Oral Medicine, General University Hospital in Prague, 128 00 Prague, Czech Republic; 6 Institute of Animal Physiology and Genetics of the CAS, v.v.i., 142 20 Prague, Czech Republic; 7 Clinic of Dentistry, St. Anne’s Faculty Hospital, 656 91 Brno, Czech Republic; 8 Clinic of Dentistry, Faculty of Medicine, Masaryk University, 625 00 Brno, Czech Republic; 9 Institute of Experimental Medicine of the CAS, v.v.i., 142 20 Prague, Czech Republic; 10 Institute of Immunology and Microbiology, First Faculty of Medicine, Charles University and General University Hospital in Prague, 128 00 Prague, Czech Republic

**Keywords:** microbiome, mycobiome, oral mucosa

## Abstract

Recurrent aphthous stomatitis (RAS) is the most common disease of the oral mucosa, and it has been recently associated with bacterial and fungal dysbiosis. To study this link further, we investigated microbial shifts during RAS manifestation at an ulcer site, in its surroundings, and at an unaffected site, compared with healed mucosa in RAS patients and healthy controls. We sampled microbes from five distinct sites in the oral cavity. The one site with the most pronounced differences in microbial alpha and beta diversity between RAS patients and healthy controls was the lower labial mucosa. Detailed analysis of this particular oral site revealed strict association of the genus *Selenomonas* with healed mucosa of RAS patients, whereas the class Clostridia and genera *Lachnoanaerobaculum*, *Cardiobacterium*, *Leptotrichia*, and *Fusobacterium* were associated with the presence of an active ulcer. Furthermore, active ulcers were dominated by *Malassezia*, which were negatively correlated with *Streptococcus* and *Haemophilus* and positively correlated with *Porphyromonas* species. In addition, RAS patients showed increased serum levels of IgG against *Mogibacterium timidum* compared with healthy controls. Our study demonstrates that the composition of bacteria and fungi colonizing healthy oral mucosa is changed in active RAS ulcers, and that this alteration persists to some extent even after the ulcer is healed.

## 1. Introduction

Recurrent aphthous stomatitis (RAS) is one of the most common diseases of the oral mucosa [1]. It is estimated that approximately a quarter of the general population may suffer from RAS [2]. It can affect otherwise healthy individuals or manifest as a comorbidity of many inflammatory diseases [3]. The aphthae are typically located on the buccal and labial mucosa and tongue, and are characterized by dissolved circular or oval ulcers with circumscribed margins and floors slightly descended below the level of the surrounding mucosa [4]. The ulcers are covered by a white fibrous coating, which contrasts with the reddish edge [1]. To date, no adequate therapy is available and patients suffer from the discomfort caused by painful ulcerations. The etiology of RAS remains unknown, but it is generally accepted that immunological, genetic, and environmental factors of local and systemic origin are involved [4,5]. Changes in microbiota composition have been identified as one of the important environmental factors [6].

The oral cavity is the main point of entry to the human body, continuously exposed to the external environment. From this rich microbial biotope, colonizing microbes spread to other body sites. Different oral niches, represented by hard tissues, such as teeth, and soft tissues, such as mucosa, and their interfaces result in a highly heterogeneous microbial composition in the oral cavity. Kim and colleagues have described that decreased abundance of *Streptococcus salivarius* and increased abundance of *Acinetobacter johnsonii* are linked to RAS incidence [7]. *Helicobacter pylori* infection has also been implicated in the disease etiopathogenesis [8,9]. *H. pylori* eradication therapy in RAS patients positively correlated with increased levels of blood vitamin B12 and healing of the ulcers [10]. Streptococci, particularly their heat-shock proteins, may also be involved in the disease pathogenesis by eliciting proliferation of autoreactive T cells that induce the immunopathological reaction [11]. Bacterial involvement in RAS thus seems to be well established; however, mycobiota composition in the oral cavity of RAS patients has not been studied as yet.

Active host defense against bacterial or fungal pathogens involves an antibody response, and elevated levels of antibodies against specific bacteria or fungi can signal chronic failure to control the pathogen. This has been recognized in patients suffering from chronic periodontitis, who have significantly elevated serum and salivary IgG and IgA levels against *Aggregatibacter actinomycetemcomitans* compared with healthy subjects [12]. Therefore, we investigated the association between elevated serum IgG or IgA antibodies to selected bacterial and fungal species recently implicated in the pathogenesis of RAS or other inflammatory oral diseases [12].

Here, for the first time, we conducted a comprehensive study mapping the overall composition of bacterial and fungal communities in patients with recurrent aphthous stomatitis, comparing them with healthy controls. Additionally, we sampled patients at the ulcer site, around the ulcer, and at a contralateral healthy site (all referred to as Active cohort) to get an overall picture of microbiota composition in the mouth. To compare the situation in relapse and remission status, we also sampled RAS patients without any active ulcers at the time of sampling (referred to as Passive cohort). Moreover, we investigated whether RAS patients have elevated serum antibodies against selected bacteria and fungi that could be associated with active disease or its remission.

## 2. Materials and Methods 

### 2.1. Patients and Sample Collection

Patients diagnosed with RAS according to Ship et al. [13] criteria were recruited at the Institute of Dental Medicine, Department of Oral Medicine, General University Hospital in Prague or at the Clinic of Dentistry, Institution Shared with St. Anne’s Faculty Hospital, Faculty of Medicine, Masaryk University, Brno, Czech Republic. All patients suffering from diseases with oral symptoms, such as food allergy, celiac disease, or autoimmune disorders, were excluded from the study. Altogether, 44 patients with RAS (23 females, 21 males) and 13 healthy controls (6 females, 7 males) were recruited. The average age ± standard deviation was 36.8 ± 12.9 years for patients and 37.8 ± 10.1 for healthy controls. Characteristics of participants, e.g., sampling site, gender, and age, are summarized in the Supplementary Material (Supplementary Tables S1 and S2). Disease state of RAS patients (Active or Passive state) included in detailed analysis of lower labial mucosa, their gender and age, and relevant characteristics of healthy controls are summarized in Supplementary Table S3.

The study was approved by the Committees for Ethics of General University Hospital and First Faculty of Medicine, Charles University, Prague (53/14; approve date 19/6/2014), Masaryk University, Faculty of Medicine (39/2015; approve date 23/6/2015), and St. Anne’s Faculty Hospital Brno (8G/2015; approve date 13/5/2015). All participants signed informed consent forms. For analysis of microbiota composition, swab samples were taken by an accredited dentist or immunologist. Fasted patients with active ulcers were sampled from the area of ulceration (Act_A), around the ulceration (aphthous surroundings; Act_AS), and from a contralateral unaffected site (contralateral healthy site; Act_CHS) (Active RAS cohort). Matching samples from healed mucosa after ulceration (Passive RAS cohort) and from healthy controls were obtained. Swabs from all three cohorts were taken from five oral sites, namely, lower labial mucosa, lower jaw mucosa, tongue, upper jaw mucosa, and buccal mucosa. Swab samples were taken using flocked swabs (FLOQSwabs™ COPAN Diagnostics Inc., 26055 Jefferson Avenue Murrieta, CA, USA), soaked in sterile SCF-1 buffer (50 mM Tris buffer (pH 7.6), 1 mM EDTA (pH 8.0), 0.5% Tween 20), as previously published [14]. Samples were stored in 400 µL of SCF-1 buffer and were immediately frozen at −80 °C. For additional information on RAS patients and healthy controls, see Supplementary Tables S1–S3.

Venous blood from the antecubital vein was collected using Vacutainer tubes (Sarstedt, Nümbrecht, Germany). After clotting, blood was centrifuged at 4000 rpm for 30 min at room temperature. Serum was collected and immediately frozen at −80 °C. To obtain saliva samples, patients chewed on a dental cotton roll (Sarstedt, Nümbrecht, Germany) for 60 s in order to stimulate salivation. Then, a collecting tube with the roll was centrifuged for 2 min at 1500 rpm. A protease inhibitor cocktail (cOmplete™, Mini, EDTA-free Protease Inhibitor Cocktail, Roche, Penzberg, Germany) was added to collected saliva, according to the manufacturer’s instructions, and the samples were frozen at −80 °C.

### 2.2. Humoral Response to Bacterial and Fungal Antigens

#### Preparation of Lysates

Pure cultures of selected bacteria were obtained from DSMZ (Deutsche Sammlung von Mikroorganismen und Zellkulturen GmbH, Braunschweig, Germany). The strains were cultured as recommended, specifically, *Acinetobacter johnsonii* (DSM 6963) overnight at 28 °C under aerobic conditions in the medium 220. CASO AGAR (Merck, Darmstadt, Germany); *Fusobacterium nucleatum* subsp. *vincentii* (DSM 19507) for 14 days at 37 °C under anaerobic conditions in the medium 78. CHOPPED MEAT MEDIUM (DSMZ, Leibniz Institute, Braunschweig, Germany); and *Mogibacterium timidum* (DSM 3998) for 7 days at 37 °C under anaerobic conditions in medium 78. CHOPPED MEAT MEDIUM (DSMZ, Leibniz Institute, Braunschweig, Germany).

Pure culture of *Candida albicans* was isolated from stool samples and determined by paired-end Sanger sequencing of ITS1 (100% identical in 551 bp with GenBank KP131671.1). *C. albicans* was cultured for 4 days at 37 °C under aerobic conditions on Malt extract agar made with 4% malt extract (Sladovna Bruntal, Bruntal, Czech Republic) and 1.5% agar (Dr. Kulich Pharma, Hradec Kralove, Czech Republic). *Candida tropicalis* was obtained from The National Institute of Public Health (NIPH, Prague, Czech Republic); it was isolated from bronchoalveolar lavage and determined by auxanogram [15].

After cultivation, bacterial and fungal cultures were centrifuged (13,500 rmp, 20 min, 4 °C), pellets were collected and washed three times with distilled water. Washed bacterial cultures were kept on ice, and afterwards subjected to pressure of 1500 psig using a French Press machine (SLM Instruments FA-078 French Pressure Cell Press, American Laboratory Trading, East Lyme, CT, USA). The lysing procedure for bacteria was repeated three times as previously described [16]. Washed fungal cultures were lysed by bead-beating four times in Lysing Matrix 210 Y tubes using FastPrep homogenizer for 60 s at 6.5 m/s (both MP Biomedicals, Irvine, CA, USA). All lysates were freeze-dried in a freeze-drier (LYOVAC GT 2, Leybold Heraeus, Cologne, Germany) and stored in aliquots at −28 °C.

### 2.3. ELISA

For ELISA, plates (96F NuncTM MaxiSorpTM, Thermo Fisher Scientific, Waltham, MA, USA) were coated overnight with lysates at concentrations of 1 g/L for bacteria and 1 mg/L for fungi. Plates were thoroughly washed with a wash buffer (1×PBS with 0.05% Tween 20) after every incubation step. Subsequently, each well was blocked with 1% BSA in PBS for 1 h. After washing, patients’ sera were applied for 2 h (dilution 1:200 for bacteria; and 1:800 for antifungal and 1:3200 for antifungal IgG. Subsequently, anti-IgG and anti-IgA secondary antibodies in a dilution of 1:2000 and 1:3000, respectively, were applied for 2 h (for bacteria, anti-Hu IgG-HRP antibody γ chain and anti-Hu IgA α chain (AFF) PEROX; both from The Binding Site Ltd., Birmingham, UK; and for fungi, Peroxidase AffiniPure F(ab’) Fragment Goat Anti-Human IgG and Peroxidase AffiniPure F(ab’) Fragment Goat Anti-Human Serum IgA, α chain specific; both from Jackson ImmunoResearch Europe Ltd., Cambridge House, St. Thomas Place, UK). Finally, a substrate solution was added (3, 3′, 5, 5′-tetramethylbenzidine in citrate buffer with H_2_O_2_; Merck, Darmstadt, Germany) and the plates were incubated for 5 min in the dark. Optical density was measured at 450 nm and 650 nm on a spectrophotometer (Multiskan Ascent Plate Reader, Lab Systems, Labsystems Diagnostics Oy, Vantaa, Finland). To compare the levels, one reference serum was applied as a reference on each ELISA plate and its mean value of OD (450–650 nm) was used as an arbitrary unit (1AU).

### 2.4. Analysis of Microbiota Composition

#### 2.4.1. DNA Extraction

Extraction of total DNA was done using the DNeasy PowerBiofilm Kit (Qiagen, Hilden, Germany), according to the manufacturer’s protocol. Thawed swab samples were thoroughly vortexed, nipped off swabs were aseptically removed, and samples were centrifuged for 10 min at 13,000 rpm. The precipitate was used for DNA isolation, including homogenization of the sample with beads (6.5 m/s, 1 min, Fast Prep, MP Biomedicals, Irvine, CA, USA) and omitting the incubation of the bead tubes at 65 °C for 5 min. 

#### 2.4.2. PCR Amplification

Total extracted DNA was used for high throughput sequencing (MiSeq platform, Illumina, San Diego, CA, USA) of the bacterial 16S rRNA gene or fungal ITS1 region. We amplified the V3–V4 region of 16S rRNA gene using degenerate barcoded primers 341F (5′-CCTACGGGNGGCWGCAG-3′) and 806R (5′-GGACTACHVGGGTWTCTAAT-3′), and the ITS1 region using degenerate fungal primers ITS1-5.8Sfw (5′-CTGTAAAAGTCGTAACAAGGTTTC-3′) and ITS1-5.8Srv (5′-AAGTTCAAAGAYTCGATGATTCAC-3′). PCR amplifications (KAPA 2G Robust Hot Start DNA Polymerase, Kapa Biosystems, Hoffmann-La Roche, Switzerland) were carried out with 25 or 27 cycles, respectively. PCR products were purified and normalized with the SequalPrep™ Normalization Plate Kit (Thermo Fisher Scientific, Waltham, MA, USA). Triplicates of the amplicons were pooled, gel eluted (NucleoSpin gel clean-up, Macherey-Nagel, Düren, Germany), and ligated with sequencing adapters (TruSeq DNA PCR-free LT Sample Preparation Kit, Illumina; KAPA HyperPlus kit, Kapa Biosystems, Hoffmann-La Roche, Switzerland). Amplicon libraries were pooled in equimolar concentrations, validated by the KAPA Library Quantification Kit (Illumina, San Diego, CA, USA), and sequenced on the MiSeq platform using the 2 × 300 bp kit at the CEITEC Genomics Core Facility (CEITEC, Masaryk University, Brno, Czech Republic). 

#### 2.4.3. Sequencing Data Analysis 

The amplicon sequencing data were processed with SEED v2.1 [17]. Paired-end reads were joined using fastq-join [18]. In fungal data, the ITS region was extracted using ITSx [19]. Chimeric sequences were detected using the UCHIME algorithm, deleted and clustered using UPARSE at a 97% similarity level in USEARCH 8.1.1861 [20]. The most abundant sequences were chosen as one representative strain per cluster. The 8,451,285 sequences of bacterial 16S rRNA were clustered into 2,488 different operational taxonomic units (OTUs), determined at a 97% similarity threshold after excluding singletons. Taxonomic classification was based on the bacterial 16S rRNA database eHOMD, version 15.1 (doi: 10.1128/mSystems.00187-18). Fungal read clustering was based on 1,648,130 sequences and resulted in 1780 OTUs at a 97% similarity threshold after excluding singletons. Fungal taxonomy was assigned using the UNITE Community (2017) [21]. Raw demultiplexed sequencing data, with sample annotations, were deposited in the NCBI database under the access code PRJNA521448. Singletons were excluded from all analyses. Alpha diversity within samples was determined using richness (number of OTUs in the sample), Chao1 and Shannon indices, and evenness after subsampling to the same sequence depth in each sample. Beta diversity, or the differences between microbial communities, were ordinated and plotted using principal coordinate analysis (PCoA). Statistical significance was assessed using Permutational Multivariate Analysis of Variance (PERMANOVA) and Analysis of Similarities (ANOSIM). To determine the discriminative features for bacterial and fungal taxonomic profiles of communities, the LEfSe analysis tool was employed (unique identifier OMICS_07818) [22].

#### 2.4.4. Bacteria–Fungi Correlation

We explored co-occurrence of bacterial and fungal OTUs using the Markov Chain Monte Carlo (MCMC) simulation model implemented in the R package BaNOCC (Schwager et al. 2017). The analyses were conducted separately for the Active, Passive, and Control cohorts. Our initial simulation for all OTUs exhibited frequent spurious correlations. This was caused particularly by rare OTUs. Consequently, only OTUs represented by at least 1% of reads in at least three samples were included in the final simulations to resolve this issue. The final simulations were run using eight independent MCMC chains, where the number of iterations was set at 500, with burn-in period and thinning interval set at 100 and 10 steps, respectively. Model convergence was checked using the Rhat statistic. Correlation coefficients deviating from zero (*p* < 0.05) were visualized using heatmaps.

## 3. Results

### 3.1. Sampling Site and Disease Status Have an Effect on the Observed Microbiota Diversity

The oral cavity harbors many distinct microbial communities [14]. This diversity stems from the oral cavity heterogeneity and the interrelationships between its different anatomic structures [23]. Thus, we focused on the overall composition of oral microbiota isolated from five distinct sites in the oral cavity, namely, lower labial mucosa (LL), lower jaw mucosa (LM), tongue (T), upper jaw mucosa (UM), and buccal mucosa (CH). To detect possible interference of different niche competitors, we focused on both bacterial and fungal communities. 

Bacterial alpha diversity at all five sites of oral cavity together was characterized by significantly more OTUs (Number of OTUs) with a higher, yet not significantly so, number of rare OTUs (Chao1) in Active and Passive RAS cohorts over healthy controls. Abundance and even distribution of species (Shannon index), as well as Evenness index alone, were similar in both cohorts (Figure S1). When we looked at the sampling sites alone, we found that the sampling site (LL, LM, T, UM, CH) and the disease status of an individual, e.g., Active or Passive RAS, healthy control, both have a meaningful effect on the observed beta diversity of bacterial communities (Figure S2). Fungal alpha diversity indices did not reveal any significant differences between Active RAS, Passive RAS, or healthy controls at all five sampled sites of oral cavity (Figure S3). Fungal beta diversity was, however, strongly impacted by the sampling site, similarly to bacterial diversity (Figure S4). 

### 3.2. Descriptive Analysis of Bacterial Microbiota in the Oral Cavity of RAS Patients with Special Emphasis on the Lower Labial Mucosa

**Figure 1:**
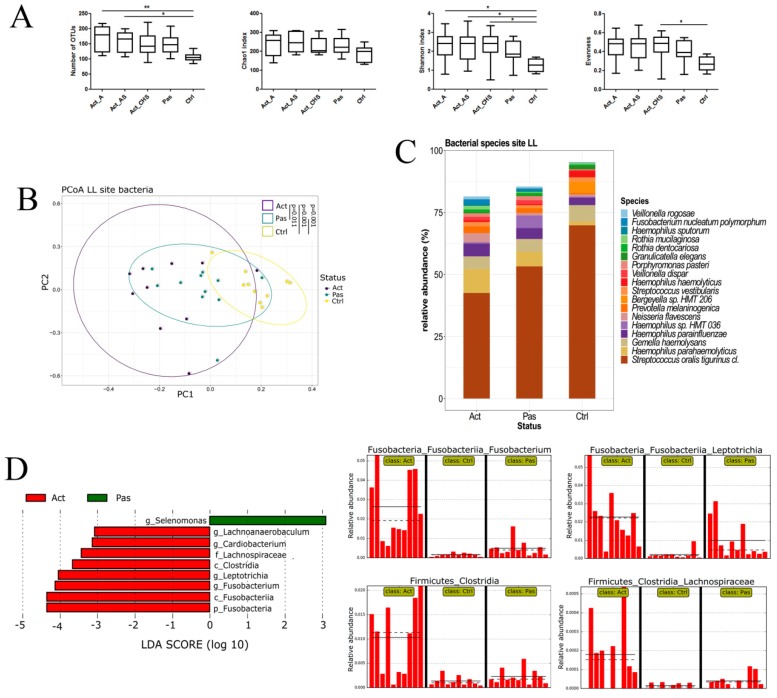
Bacterial abundance and diversity in samples of Active and Passive recurrent aphthous stomatitis (RAS) patients and healthy controls from lower labial mucosa (LL). (**A**) Alpha diversity expressed as the number of operational taxonomic units (OTUs), Chao1 index, Shannon index, and Evenness at the site of ulceration (Act_A), at the site around ulceration (Act_AS), at a contralateral unaffected site of the oral cavity of RAS patients (Act_CHS), in RAS patients without manifested ulcer (Pas) and in healthy controls (Ctrl). (**B**) Beta diversity of the LL site is presented in the form of PCoA plot. (**C**) Taxonomic profile of the bacterial community at the species level shows the most abundant bacterial species in RAS patients at the site of ulceration (Act_A), in RAS patients without manifested ulcer (Pas), and in healthy controls (Ctrl). (**D**) LEfSe (LDA Effect Size) algorithm revealed increased levels of some genera in patients with RAS. Significant differences are denoted by * *p* < 0.05 and ** *p* < 0.01.

The most pronounced differences between healthy controls and Active and Passive patients were found in the lower labial mucosa (LL); therefore, we further focused on the LL in our detailed analysis. RAS patients were found to have an increased alpha diversity over healthy controls, not only at the ulceration site, but also in other tested areas of the oral cavity, i.e., around the ulcer and at the contralateral unaffected site (Figure 1A). Beta diversity in both the Active and Passive RAS patients was significantly more similar to each other than to healthy controls (Figure 1B). Taxonomic profile of the bacterial community revealed the highest abundance of *Streptococcus oralis* in healthy controls, further decreasing in Passive and Active RAS patients (Figure 1C); the same finding was obtained also in other sampled areas of oral cavity (see Figure S2). LEfSe analysis focused on disease status unveiled discriminative association of the genera *Lachnoanaerobaculum*, *Cardiobacterium*, *Leptotrichia*, *Fusobacterium*, and the class *Clostridia* with Active RAS patients, whereas the genus *Selenomonas* was significantly associated with Passive RAS patients (Figure 1D).

### 3.3. Descriptive Analysis of Fungal Microbiota in the Oral Cavity of RAS Patients with Special Emphasis on the Lower Labial Mucosa

**Figure 2:**
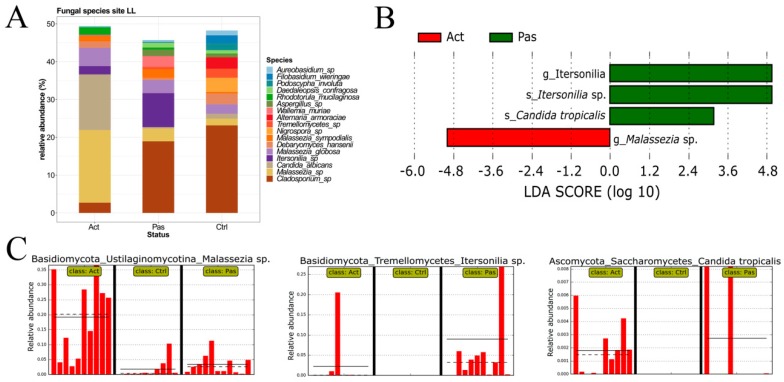
Fungal composition in samples from the lower labial mucosa. (**A**) Relative abundance of the most abundant fungal species from the lower labial mucosa (LL) of RAS patients at the site of ulceration (Act), RAS patients without manifested ulcer (Pas), and healthy controls (Ctrl). (**B**,**C**) LEfSe analysis revealed a significant association of *Malassezia* sp. with Active patients and species *Itersonilia* sp. and *Candida tropicalis* with Passive RAS patients.

Similarly to the analysis of bacterial composition, we evaluated the distribution of fungal communities in samples from the LL. Fungal taxonomic profile at the species level revealed that *Cladosporium* sp. dominated in healthy controls and in the Passive cohort, where it was accompanied by *Itersonilia* sp., whereas *Malassezia* sp. and *Candida albicans* were highly enriched in the Active RAS cohort (Figure 2A). LEfSe analysis additionally found *Malassezia* spp. to be significantly associated with the Active cohort. Furthermore, the species *Itersonilia* and *Candida tropicalis* were found to be discriminative features for Passive RAS patients; however, they were not observed in the whole cohort, but rather in a few individuals (Figure 2B,C). 

### 3.4. Bacterial and Fungal Correlation Networks in RAS

**Figure 3:**
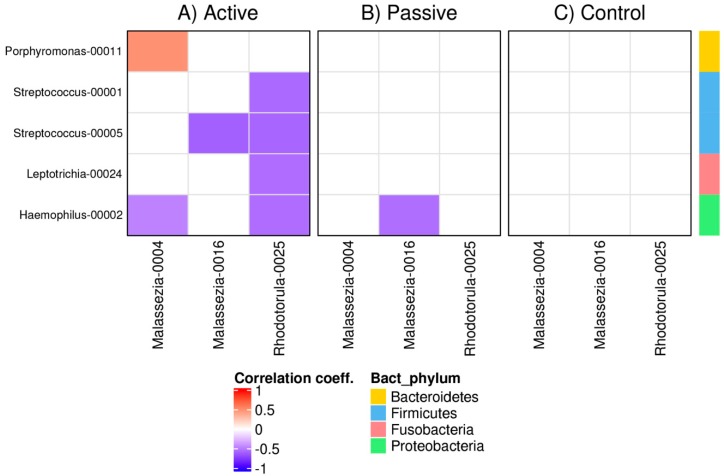
Correlation networks between bacterial and fungal OTUs in RAS patients compared with healthy controls. Correlation pattern between bacterial and fungal OTUs in (**A**) Active, (**B**) Passive, and (**C**) Control cohort. Blue squares indicate inverse correlations (negative values) and red squares indicate positive correlations (positive values) in proportions of reads between individual OTUs. Color intensity shows the magnitude of the association; the darker the color, the stronger the association. Correlations that did not deviate from zero (*p* < 0.05) are indicated by white color. Statistical significance is based on Markov Chain Monte Carlo sampling.

We investigated co-occurence/co-avoidance between bacterial and fungal OTUs in the oral cavity of RAS patients and healthy controls. Highly supported correlations were identified for OTUs of the bacteria *Porphyromonas*, *Streptococcus*, *Leptotrichia*, and *Haemophilus*, interacting with the fungi *Malassezia* and *Rhodotorula*. Using microbial and fungal databases, the OTUs were identified as: OTU00011 *Porphyromonas pasteri*; OTU00001 *Streptococcus oralis*; OTU00005 *Streptococcus vestibularis*; OTU00024 *Leptotrichia* HMT; OTU00002 *Haemophilus prainfluenzae*; OTU0004 *Malassezia* sp.; OTU0016 *Malassezia globosa*; OTU0025 *Rhodotorula mucilaginosa*. Active ulcers were dominated by *Malassezia* spp., one of which was found to have a negative correlation with *Haemophilus* and a positive correlation with *Porphyromonas* OTUs. The other *Malassezia* had a negative correlation with *Streptococcus* in active ulcers, but not in healed mucosa. However, a negative correlation of this particular *Malassezia* OTU with a *Haemophilus* OTU was observed in healed mucosa. *Rhodotorula* had negative associations with both *Strepococcus* OTUs, *Leptotrichia* and *Haemophilus* in the Active cohort. None of the observed associations were found in healthy controls (Figure 3).

### 3.5. Serum Levels of IgG Against Mogibacterium timidum are Elevated in Patients with RAS

**Figure 4:**
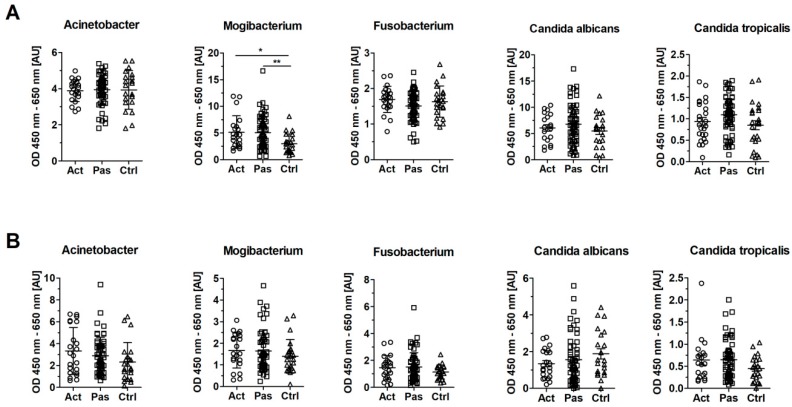
Serum levels of IgG and IgA antibodies in RAS and healthy cohort. (**A**) Levels of IgG against selected bacteria and fungi in serum from patients with active (Act) and passive (Pas) stage of RAS and healthy controls (Ctrl). (**B**) Levels of IgA against selected bacteria and fungi in serum from patients with Active (Act) and Passive (Pas) stage of RAS and healthy controls (Ctrl). Each point represents a sample from one patient or healthy control. Statistical significance was confirmed using Kruskal–Wallis test with Dunn’s multiple comparison test. Significant differences are denoted by * *p* < 0.05 and ** *p* < 0.01. Reference serum was added to each ELISA plate and its mean value of OD (450–650 nm) was used as an arbitrary unit (1 AU).

To analyze humoral response to bacteria and fungi, we measured serum levels of antibodies against bacteria *Acinetobacter johnsonii*, *Fusobacterium nucleatum*, and *Mogibacterium timidum* and fungi *Candida albicans* and *Candida tropicalis* in patients with Active or Passive stage of RAS and in healthy controls. In the sera from patients in both the Active and Passive stage of RAS, we found significantly increased levels of IgG against *M. timidum* over healthy controls (*p* = 0.015 and 0.003, respectively) (Figure 4A). RAS patients did not significantly differ from healthy controls in levels of IgG against *A. johnsonii* and *F. nucleatum* or against *C. albicans* and *C. tropicalis*, although abundance of *C. tropicalis* was increased in RAS patients (Figure 2B). Serum levels of specific IgA against any of the tested microbes did not show any significant changes. We only observed a tendency towards an increased anti-*C. albicans* IgA response in the Control cohort, and the opposite trend in IgA response against all tested bacteria and *C. tropicalis* in the same cohort (Figure 4B).

## 4. Discussion

To date, only a few studies have examined the specifics of microbiota composition in patients with recurrent aphthous stomatitis [6,7,24]. In agreement with these previous studies, we observed that microbiota in RAS patients differs from microbiota found in healthy individuals. We sampled patients’ microbiota either from minor RAS lesions or from healed mucosa at five different sites of the oral cavity. Lower labial mucosa showed the most pronounced differences between Active or Passive RAS patients and healthy controls, which is why we focused on this particular oral site in detailed analyses. Consistent with the published literature, inflammation, i.e., the presence of ulcer, was found to be the main determining factor affecting microbiota composition [6]. Moreover, we showed that not only microbiota within the ulcer itself, but also in the surrounding area differ from healthy controls.

As a pioneer colonizer and the most represented bacterial genus in the oral cavity, *Streptococcus* belongs to the core of healthy oral microbiome [25]. We noticed that its abundance decreased during active ulcer manifestation and increased a little after ulcer healing. Furthermore, the abundance of *S. oralis* was slightly higher in healthy controls (69.8%) over RAS patients (42.6% in the Active cohort and 50.9% in the Passive cohort). *Streptococcus* populations may fluctuate in response to a disturbed environment, which is in line with earlier observations [7,26]. A lower abundance of *Streptococcus*, together with a higher abundance of *Acinetobacter johnsonii*, has been recently linked to a higher risk of RAS [7]. In agreement with this study, we also found a higher abundance of *A. johnsonii* in RAS patients over healthy controls (0.031% in the Active cohort; 0.002% in the Passive cohort; and 0.001% in healthy controls), although not significantly so. An initial imbalance in bacterial microbiota might be crucial for expansion of opportunistic pathogens, consequently leading to compositional changes in fungal microbiota and further to the disruption of oral tissue through inter-kingdom interaction [27,28]. A greater amount of *Haemophilus parahaemoliticus* or *H. parainfluenzae* found in RAS patients over healthy controls further strengthens the connection of opportunistic pathogens with the risk of RAS. In addition, opportunistic pathogens *Fusobacterium* or *Leptotrichia*, whose abundance we found to be elevated in the Active RAS cohort, have been described as part of the normal oral microbiota [29,30]. *Fusobacterium nucleatum* has been previously connected to incidence of dental caries and periodontitis [31,32]. Furthermore, *F. nucleatum* can spread to the intestinal tract and is tightly associated with colorectal carcinoma development [33]. The most abundant fungal species in the Active RAS cohort belong to the genus *Malassezia*, which has been mainly reported as a commensal and opportunistic pathogen of the skin, but nowadays is being included also as a member of the oral cavity mycobiome [34]. Using the software tool LEfSe [35], we discovered the genus *Malassezia* to be highly enriched in patients with an active RAS ulcer, which is consistent with the hypothesis of microbial shift in favor of opportunistic pathogens. This observation is also in line with published data connecting the presence of *Malassezia* with incidence of other diseases, including dandruff, seborrheic dermatitis, and psoriasis [36,37,38,39,40]. The second most abundant species in the Active RAS cohort was *Candida albicans*, often described either as a commensal or opportunistic pathogen in the oral cavity [34,41]. *C. albicans* is believed to get virulent by modulation of co-colonizing microbiota [28]. Consistently with the published literature, we detected *C. albicans* in low amounts as a commensal in healthy oral cavity, with the abundance rapidly increasing in active ulcers. Another two species were found only in RAS patients, namely *Itersonilia* sp. and *Candida tropicalis*. Associated mostly with healed mucosa, both species were present to some extent also at the site of active ulcers. *Itersonilia* is usually described as a plant commensal or pathogen; therefore, its presence in the oral cavity is probably inconsequential in relation to RAS, since it is most likely associated with food intake [42,43,44]. *C. tropicalis* is considered to be the second most virulent *Candida* species and a very strong biofilm producer [45]. In our dataset, we found *C. tropicalis* to be abundant rather in few individuals with healed mucosa than in the whole cohort of RAS patient.

Fascinating hints of inter-kingdom interactions led us to perform correlation analysis and, for the first time, to explore the co-occurrence network between bacteria and fungi within the oral cavity of RAS patients and healthy controls. Apart from a negative correlation of *Malassezia* sp. with *Haemophilus parainfluenzae* and its positive correlation with *Porphyromonas pasteri*, we unveiled a number of associations of *Rhodotorula mucilaginosa* with bacterial species during active RAS. Bacterial–fungal networks might be of great importance and, in the future, might potentially serve for prognostic or diagnostic purposes in RAS.

Antibody response against specific microbes is a sign of activated adaptive immunity, which could either lead to specific pathogen clearance, or result in a chronic failure to control the pathogen [31,46]. This has been documented in chronic periodontitis, where elevated levels of IgG antibodies against *Aggregatibacter actinomycetemcomitans* are connected to the severity of periodontal tissue destruction [12,47]. Therefore, we investigated serum IgG and IgA antibodies against selected bacterial and fungal species, recently connected to pathogenesis of RAS or other inflammatory oral diseases [12]. Increased presence of *Mogibacterium timidum* in the oral cavity has been previously linked to periodontitis incidence [48]. Here, we reported, for the first time, that both Active and Passive RAS patients have elevated levels of IgG antibodies against *M. timidum* as compared to healthy controls, while IgA response to all and IgG response to other analyzed microbes was unchanged. This may be caused by the fact that multiple dominant antigens from these microbes may be shared with tolerated commensals or by inability of these microbes to produce immunodominant virulence factors when cultivated in vitro in artificial growth media [49,50,51]. Moreover, microbes may respond to inflammatory conditions, such as in inflamed mucosa during RAS, by producing different surface antigens, thus differing even from isolates from healthy mucosa [52,53]. Nevertheless, by preparing these microbes in vitro under standardized conditions, we were able to compare all tested groups within a single assay. Therefore, while we cannot exclude importance of antibody response to components from the other microbes which are expressed only during the infection, our results suggest a potential role of *M. timidum* in the pathogenesis of RAS and point at an interesting biomarker.

## 5. Conclusions

To understand the role of bacterial and fungal communities in the human oral cavity in health and disease, we need to acquire thorough knowledge of their presence and interactions. We are the first to conduct a comprehensive study mapping the overall composition of bacterial and fungal communities in patients with RAS. We found increased serum levels of antibodies against *Mogibacterium* sp. in RAS patients, indicating its potential role in the pathogenesis of RAS. Our results further propose an intriguing hypothesis of altered inter-kingdom interactions, taking place especially during active RAS. We suggest that a pre-existing disruption of microbial communities (dysbiosis) might allow opportunistic pathogens to prevail, leading to oral tissue damage. Future studies would benefit from analyzing larger populations of patients and healthy controls to yield more predictive power. As we observed some interesting changes in microbial associations during active RAS, a deeper analysis of microbial metabolic functions could also reveal further factors important for disease development. 

## Supplementary Materials

The following are available online at https://www.mdpi.com/2076-2607/7/12/636/s1. Table S1: Characteristics of patients and controls in the study. Table S2: Sampling sites of patients and controls. Table S3: Characteristics of patients suffering from RAS and healthy controls, both sampled from the lower labial mucosa (LL). Figure S1: Bacterial alpha diversity in active ulcer of RAS patients compared with the same site after ulcer healing and with healthy controls at five merged sites of sampling. Figure S2: Beta diversity of bacterial communities in active ulcer of RAS patients compared with the same site after ulcer healing and with healthy controls at different sites of sampling. Figure S3: Fungal alpha diversity in an active ulcer of RAS patients compared with the same site after ulcer healing and with healthy controls in five merged sites of oral cavity. Figure S4: Beta diversity of fungal communities in an active ulcer of RAS patients compared with the same site after ulcer healing and with healthy controls at different sampling sites.
