# Supplementary material for: Oral Microbiota Composition and Antimicrobial Antibody Response in Patients with Recurrent Aphthous Stomatitis
Source: Microorganisms. 2019 Dec 1;7(12):636. doi: 10.3390/microorganisms7120636 (PMC6955784; doi:10.3390/microorganisms7120636)
Supplement: Supplementary file 1 [file microorganisms-07-00636-s001.pdf]

Supplementary Material

1. Supplementary tables and figures

1.1. Tables Related to Material and Methods

Table S1. Characteristics of patients and controls in the study.

		**Range (average ± SD)**
		**Age**
**RAS**	**female** (*n* = 23)	19–75 (36 ± 14.1)
	**male** (*n* = 21)	19–60 (37.9 ± 11.4)
Total	*n* = 44	19–75 (36.8 ± 12.9)
**Controls**	**female** (*n* = 6)	23–61 (41.8 ± 12.5)
	**male** (*n* = 7)	26–42 (34.4 ± 5.6)
Total	*n* = 13	23–61 (37.8 ± 10.1)

Abbreviations: RAS, recurrent aphthous stomatitis; SD, standard deviation.

Table S2. Sampling sites of patients and controls.

		**LL**	**LM**	**CH**	**T**	**UM**	**Total count**
RAS samples	Passive	11	4	11	8	3	**37**
	Active	10	3	9	8	3	**33**
	Total count	**21**	**7**	**20**	**16**	**6**	**70**
Control samples		10	3	10	8	3	**34**

Abbreviations: LL, lower labial mucosa; T, tongue; UM, upper jaw mucosa; LM, lower jaw mucosa; CH, buccal mucosa; RAS, recurrent aphthous stomatitis.

Table S3. Characteristics of patients suffering from RAS and healthy controls, both sampled from the lower labial mucosa (LL).

	**RAS**			
	**ID**	**State**	**Gender**	**Age**
	P1	Act	F	49
	P2	Act	M	34
	P3	Act, Pas	F	64
	P4	Act, Pas	M	28
	P5	Act	F	41
	P6	Act, Pas	M	30
	P7	Act	F	19
	P8	Act	M	31
	P9	Act	F	34
	P10	Act	M	36
	P28	Pas	F	42
	P29	Pas	M	42
	P33	Pas	F	37
	P34	Pas	M	30
	P35	Pas	F	24
	P36	Pas	M	37
	P37	Pas	F	24
	P38	Pas	M	39
Total count	18	Act (n = 10)<br>Pas (n = 11)	F (n = 9)<br>M (n = 9)	F: 19–64 (37.1 ± 13.3)<br>M: 28–42 (34.1 ± 4.7)
	**Controls**			
	**ID**	**State**	**Gender**	**Age**
	K1	-	M	34
	K2	-	M	39
	K3	-	F	37
	K4	-	M	40
	K5	-	F	45
	K6	-	F	52
	K7	-	F	61
	K8	-	M	42
	K9	-	F	23
	K10	-	M	26
Total count	10		F (n = 5)<br>M (n = 5)	F: 23–61 (43.6 ± 12.9)<br>M: 26–42 (36.2 ± 5.7)

Abbreviations: ID, identification of patients (P) and controls (K); RAS, recurrent aphthous stomatitis; Act, patient with active ulcer; Pas, patient with healed mucosa after ulceration; F, female; M, male.

1.2 Figures and Tables Related to Results

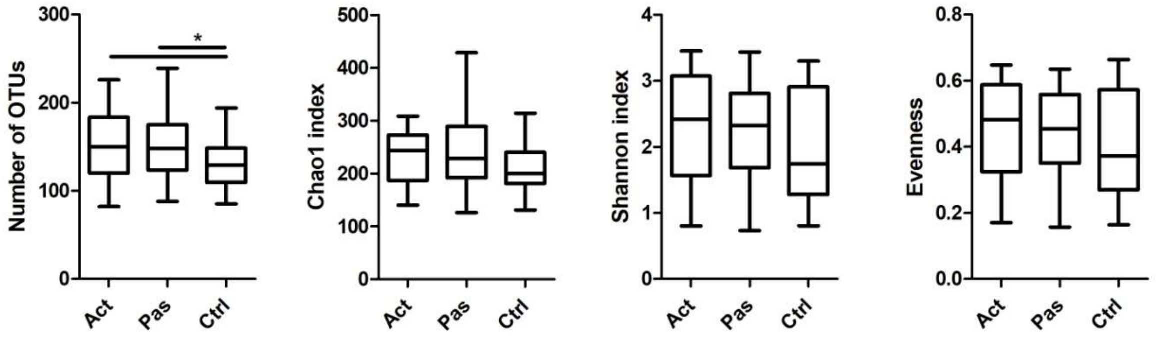

Figure S1. Bacterial alpha diversity in active ulcer of RAS patients compared with the same site after ulcer healing and with healthy controls at five merged sites of sampling. Common indicators of diversity such as Number of operational taxonomic units (OTUs), Chao1, Shannon index, and Evenness index were used to measure compositional diversity at five merged sites of oral cavity. Differences between groups were determined using ANOVA. *$p < 0.05$. Act, active ulcer of RAS patients; Pas, healed mucosa after RAS ulcer; Ctrl, healthy controls.

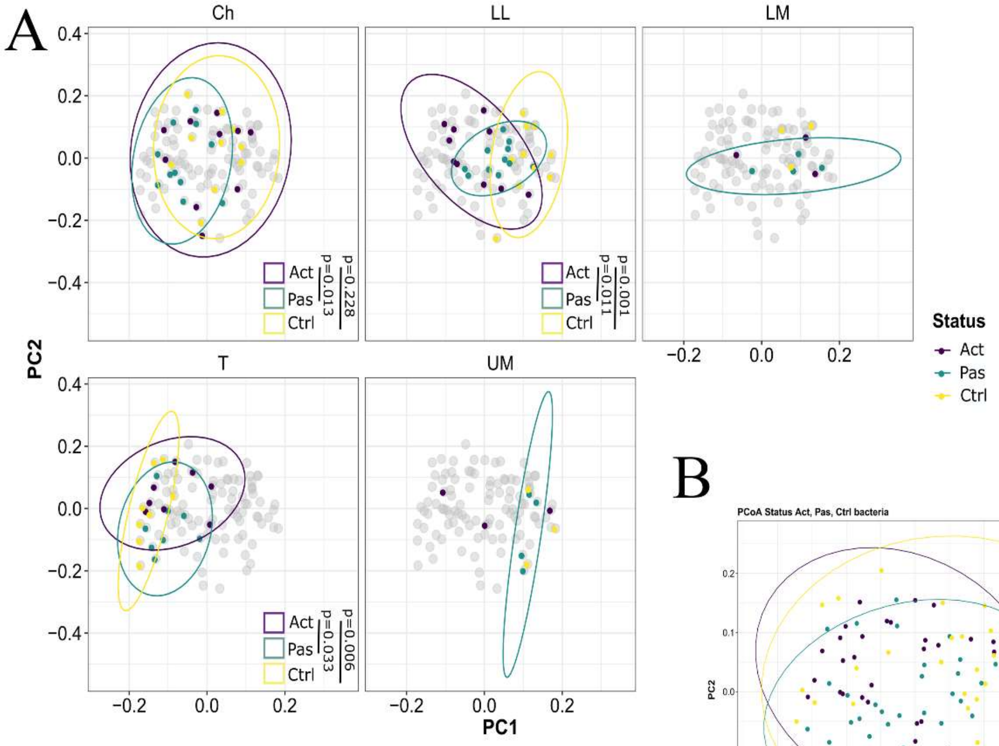

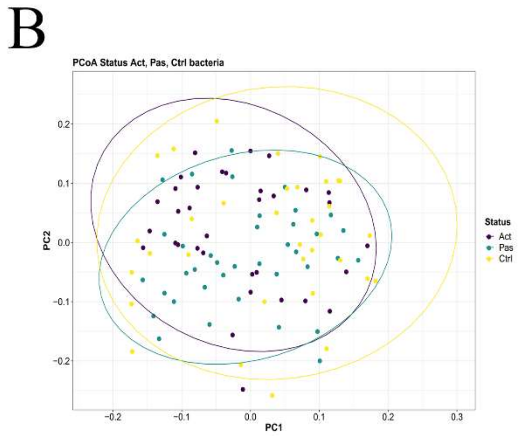

ANOSIM R				p-value			
LL	act	pas	ctrl	LL	act	pas	ctrl
act	x	0.1703	0.6127	act	x	0.011	0.001
pas	0.1703	x	0.3138	pas	0.011	x	0.001
ctrl	0.6127	0.3138	x	ctrl	0.001	0.001	x
Ch	act	pas	ctrl	Ch	act	pas	ctrl
act	x	0.1775	0.04178	act	x	0.013	0.228
pas	0.1775	x	0.2191	pas	0.013	x	0.023
ctrl	0.04178	0.2191	x	ctrl	0.228	0.023	x
T	act	pas	ctrl	T	act	pas	ctrl
act	x	0.1752	0.2919	act	x	0.033	0.006
pas	0.1752	x	0.1607	pas	0.033	x	0.055
ctrl	0.2919	0.1607	x	ctrl	0.006	0.055	x

All sites	PERMANOVA R	PERMANOVA P-val	Act+A/AS/CHS	PERMANOVA R	PERMANOVA P-val
SampleSite	0.19368	0.001	SampleSite	0.16129	0.001
Status	0.054	0.001	SampleType	0.01787	0.38

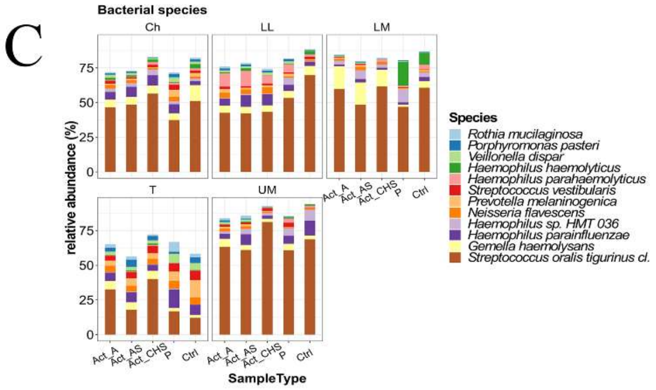

Figure S2. Beta diversity of bacterial communities in active ulcer of RAS patients compared with the same site after ulcer healing and with healthy controls at different sites of sampling. (**A**) Two-dimensional principal coordinates analysis (PCoA) was used to show distribution of heterogeneity among active ulcer, healed mucosa, and healthy controls at different sites of sampling. (**B**) Beta diversity of all individuals grouped together. Statistical significance was determined using ANOSIM and PERMANOVA. * $p < 0.05$, ** $p < 0.01$, *** $p < 0.001$. (**C**) Microbial profile at different sampling sites was determined in an active ulcer of RAS patients (Act_A), in an area around ulceration (Act_AS), at a corresponding site after ulcer healing (Pas), at a contralateral healthy site (Act_CHS) of RAS patients, and in healthy controls (Ctrl). Act, active ulcer of RAS patients; Pas, healed mucosa after RAS ulcer; Ctrl, healthy controls; CH, buccal mucosa; LL, lower labial mucosa; LM, lower jaw mucosa; T, tongue; UM, upper jaw mucosa; Act_A, active ulcer of RAS patient; Act_AS, surrounding site of active ulcer; Act_CHS, contralateral healthy site of RAS patient.

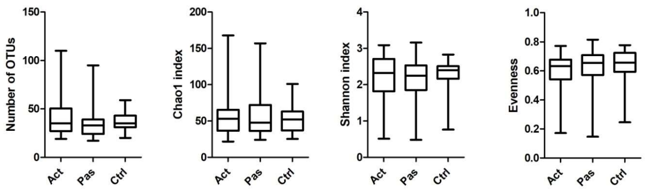

Figure S3. Fungal alpha diversity in an active ulcer of RAS patients compared with the same site after ulcer healing and with healthy controls in five merged sites of oral cavity. Common indicators of diversity such as Number of OTUs, Chao1, Shannon index and Evenness index were used to measure compositional diversity in merged sites of sampling. Differences between groups were determined using ANOVA. Act_A, active ulcer of RAS patients; Pas, healed mucosa after RAS ulcer; Ctrl, healthy controls.

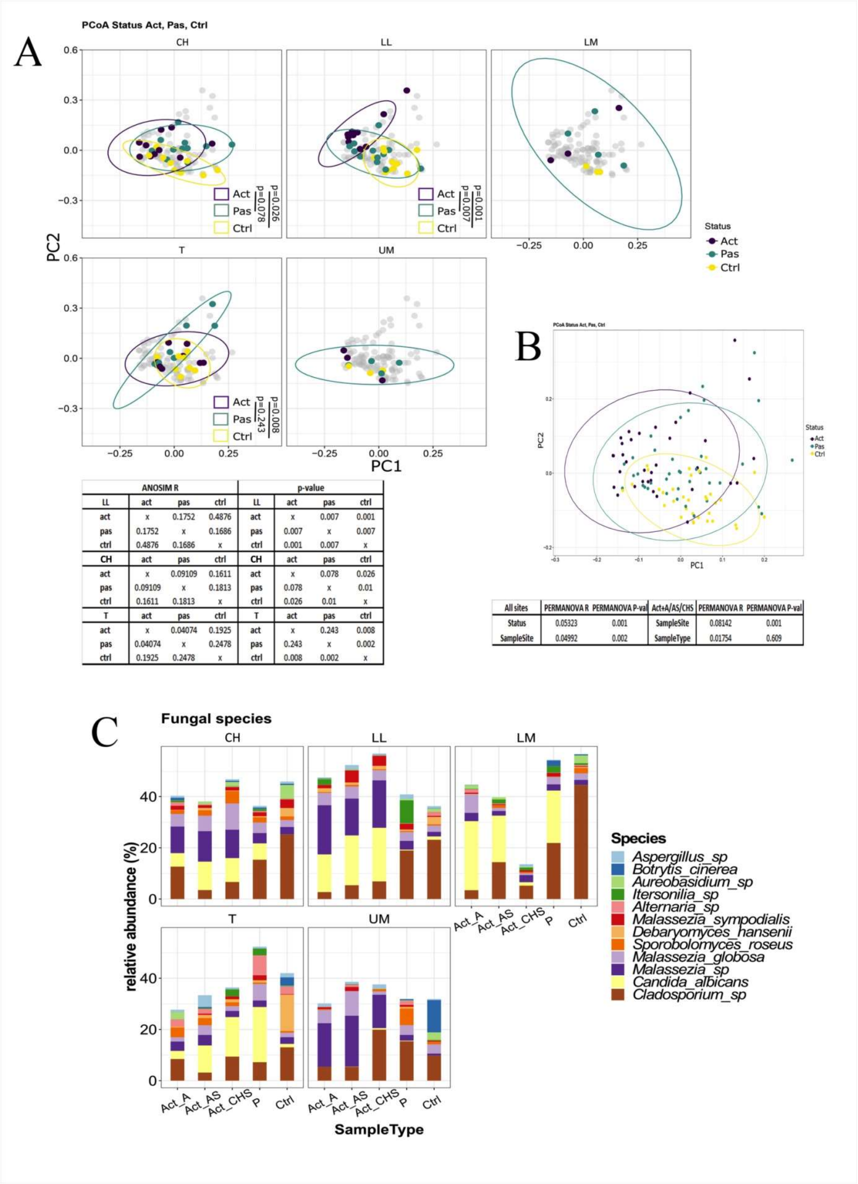

ANOSIM R				p-value			
LL	act	pas	ctrl	LL	act	pas	ctrl
act	x	0.1752	0.4876	act	x	0.007	0.001
pas	0.1752	x	0.1686	pas	0.007	x	0.007
ctrl	0.4876	0.1686	x	ctrl	0.001	0.007	x
CH	act	pas	ctrl	CH	act	pas	ctrl
act	x	0.09109	0.1611	act	x	0.078	0.026
pas	0.09109	x	0.1813	pas	0.078	x	0.01
ctrl	0.1611	0.1813	x	ctrl	0.026	0.01	x
T	act	pas	ctrl	T	act	pas	ctrl
act	x	0.04074	0.1925	act	x	0.243	0.008
pas	0.04074	x	0.2478	pas	0.243	x	0.002
ctrl	0.1925	0.2478	x	ctrl	0.008	0.002	x

All sites	PERMANOVA R	PERMANOVA P-val	Act+A/AS/CHS	PERMANOVA R	PERMANOVA P-val
Status	0.05323	0.001	SampleSite	0.08142	0.001
SampleSite	0.04992	0.002	SampleType	0.01754	0.609

Figure S4. Beta diversity of fungal communities in an active ulcer of RAS patients compared with the same site after ulcer healing and with healthy controls at different sampling sites. (**A**) Two-dimensional principal coordinates analysis was used to show distribution of heterogeneity among active ulcer, healed mucosa, and healthy controls at different sites of sampling. (**B**) Beta diversity of all individuals and sampled sites grouped

together. (**C**) Taxonomical profile of fungal species at different sites of sampling. Statistical significance was determined using ANOSIM and PERMANOVA. * $p < 0.05$, ** $p < 0.01$, *** $p < 0.001$. Act, active ulcer of RAS patients; Pas, healed mucosa after RAS ulcer; Ctrl, healthy controls; CH, buccal mucosa; LL, lower labial mucosa; LM, lower jaw mucosa; T, tongue; UM, upper jaw mucosa; Act_A, area of ulceration of RAS patient; Act_AS, surrounding area of active ulcer; Act_CHS, contralateral healthy site of RAS patient.
